# Supplementary material for: Effects of LiBF4 Addition on the Lithium-Ion Conductivity of LiBH4
Source: Molecules. 2022 Mar 28;27(7):2187. doi: 10.3390/molecules27072187 (PMC9000853; doi:10.3390/molecules27072187)
Supplement: Supplementary file 1 [file molecules-27-02187-s001.zip › molecules-1603504-supplementary.pdf]

Supporting information

Effects of $LiBF_4$ addition on the lithium-ion conductivity of $LiBH_4$

Laura M. de Kort, Valerio Gulino, Didier Blanchard, Peter Ngene*

Materials Chemistry and Catalysis, Debye Institute for Nanomaterials Science, Utrecht University, Universiteitsweg 99, 3584 DX Utrecht, Netherlands

* Corresponding authors: P.Ngene@uu.nl,

S1. Overview of all prepared samples

For the preparation of (1-*x*)$LiBH_4$ - *x* $LiBF_4$ and (1-*x*)$LiBH_4$ – *x* $Li_2B_{12}H_{12}$ the mass ratio was calculated using the molecular weight of $LiBH_4$ (21.78 g mol^{-1}), $LiBF_4$ (93.75 g mol^{-1}) and $Li_2B_{12}H_{12}$ (155.71 g mol^{-1}) and the intended molar fraction of the final product. The compositions of the samples in weight percentage and molar percentage are given in **Table S1**.

Table S1 – Composition of the samples discussed in this work in wt% and mol%

**Mixed compounds**		**Weight percentage**		**Molar percentage**	
**$LiBH_4$- x $LiBF_4$**	**$T_{reaction}$, $t_{reaction}$**	**$LiBH_4$**	**$LiBF_4$**	**$LiBH_4$**	**$LiBF_4$**
$LiBH_4$-20%$LiBF_4$	150 °C, 30 min	44.5	55.5	77.5	22.5
$LiBH_4$-20%$LiBF_4$	280 °C, 30 min	48.2	51.8	80.0	20.0
$LiBH_4$-20%$LiBF_4$	280 °C, 1 hour	48.1	51.9	80.0	20.0
**$LiBH_4$-$Li_2B_{12}H_{12}$**		**$LiBH_4$**	**$Li_2B_{12}H_{12}$**	**$LiBH_4$**	**$Li_2B_{12}H_{12}$**
$LiBH_4$-10% $Li_2B_{12}H_{12}$		55.8	44.2	90.0	10.0
$LiBH_4$-25% $Li_2B_{12}H_{12}$		30.1	69.9	75.4	24.6
$LiBH_4$-50% $Li_2B_{12}H_{12}$		12.6	87.4	50.7	49.3
$LiBH_4$-75% $Li_2B_{12}H_{12}$		4.6	95.4	25.5	74.5

S2. Conductivity of $LiBH_4$-$LiBF_4$ and $LiBH_4$-$Li_2B_{12}H_{12}$

The conductivity of the $LiBH_4$-$LiBF_4$ and $LiBH_4$-$Li_2B_{12}H_{12}$ mixtures is based on Nyquist plots, as shown in **Figure S1**. For these exemplary Nyquist plots, the following parameters were obtained from a least squares fit of the given equivalent circuit consisting of a resistance (R) and constant phase element (with $CPE(Q,n) = (Q\cdot(i\omega)^n)^{-1}$):

$LiBH_4$ – 20% $LiBF_4$ – $R = 2.32 \cdot 10^3\ \Omega$, $Q = 4.33 \cdot 10^{-9}\ Ss^n$, $n = 0.90$

$LiBH_4$ – 25% $Li_2B_{12}H_{12}$ – $R = 1.76 \cdot 10^4\ \Omega$, $Q = 2.06 \cdot 10^{-9}\ Ss^n$, $n = 0.94$

While we mainly report the resistance of the studied systems in this work, similar values for Q and n are observed for the other impedance measurements.

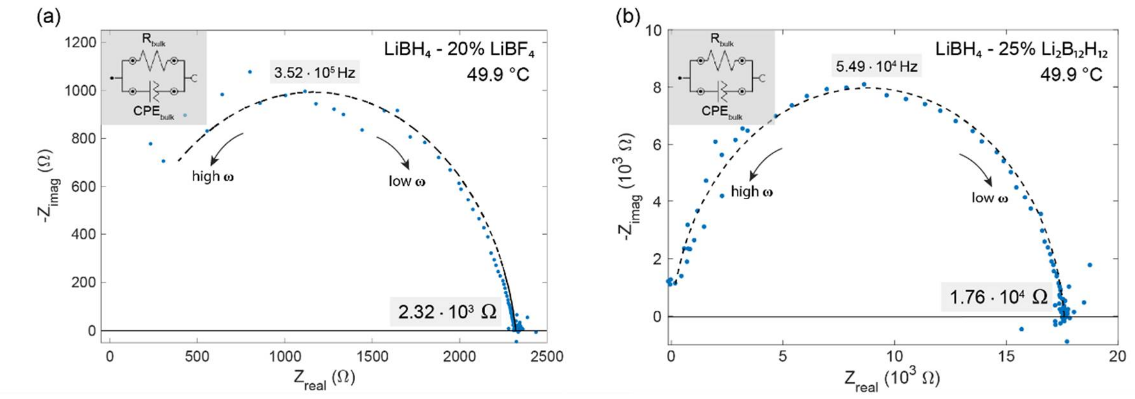

Figure S1 – Exemplary Nyquist plots obtained at 50 °C for (a) $LiBH_4$-$LiBF_4$ compound and (b) $LiBH_4$-$Li_2B_{12}H_{12}$ compound prepared 280 °C. The equivalent circuit used to fit the Nyquist plot is shown in the top left corner.

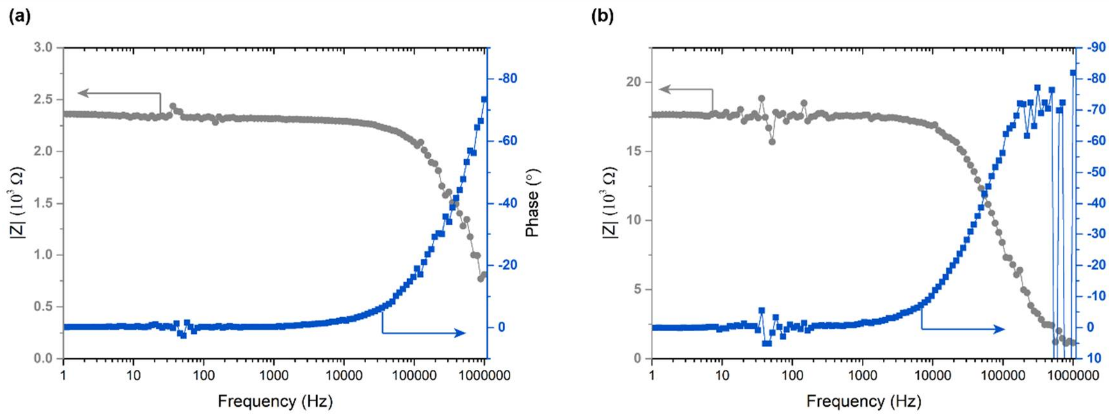

Figure S2 – Exemplary Bode plots obtained at 50 °C for (a) $LiBH_4$-$LiBF_4$ compound and (b) $LiBH_4$-$Li_2B_{12}H_{12}$ compound prepared 280 °C.

S3. Optimization of $LiBH_4$- $LiBF_4$ composition and synthesis temperature

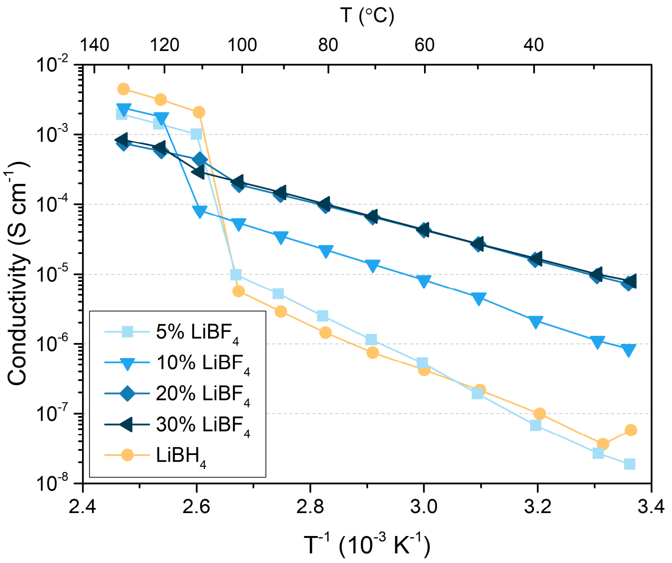

Figure S3. Arrhenius plots of $LiBH_4$-$LiBF_4$ mixtures with different composition. The plot shows that addition of 20 - 30 mol% $LiBF_4$ leads to the highest ionic conductivity at moderate temperatures. Above and below these concentrations, the conductivty at lower temperatures decreases.

The Arrhenius conductivity plots of 80% $LiBH_4$-20% $LiBF_4$ mixtures heated to different temperatures is shown on **Figure S4**. The plot shows that 280 °C gives the optimum conductivity. Increasing the reaction temperature to 300 °C did not led to further improvement on the ionic conductovity of the resulting samples.

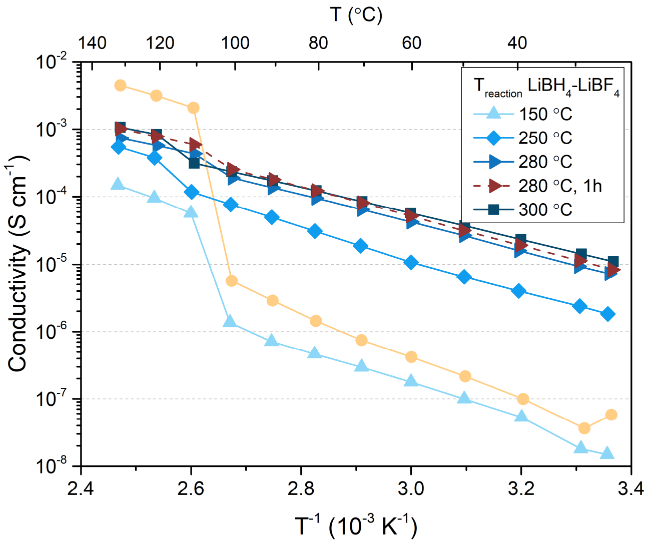

Figure S4. Arrhenius plots of $LiBH_4$-$LiBF_4$ mixtures synthesized at different temperatures.

S4. Optimization of $LiBH_4$-$Li_2B_{12}H_{12}$ composition

To see which $LiBH_4$-$Li_2B_{12}H_{12}$ composition correlated best with the data obtained for $LiBH_4$-$LiBF_4$, the molar fraction was varied from 10 to 75 molar %. The corresponding DRIFTS spectra and XRD diffractograms are shown in **Figure S5**. Accordingly, with increasing $Li_2B_{12}H_{12}$ content the $Li_2B_{12}H_{12}$ peaks become more intense, while the $LiBH_4$ peaks become less intense. The DRIFTS spectrum of $LiBH_4$ - 25% $Li_2B_{12}H_{12}$ resembled the DRIFTS spectrum of the $LiBH_4$ - 20% $LiBF_4$ mixture best.

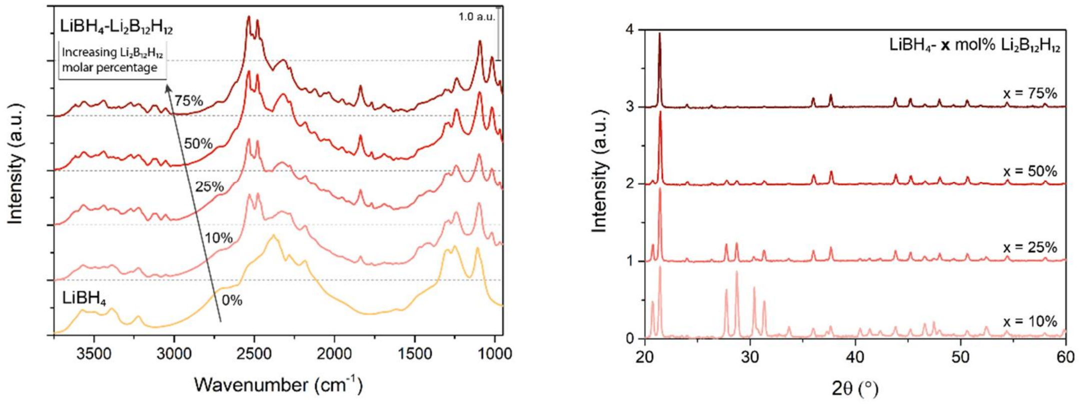

Figure S5. (left) DRIFTS spectra and (right) XRD diffraction patterns $LiBH_4$-$Li_2B_{12}H_{12}$ mixtures synthesized at 280 °C. In the DRIFTS graphs $LiBH_4$ is included for comparison.

The Arrhenius conductivity plots of these samples are shown in **Figure S6**. Based on the conductivity data of these samples, it is clear that the conductivity is optimal for a molar fraction between 25 and 50% $Li_2B_{12}H_{12}$.

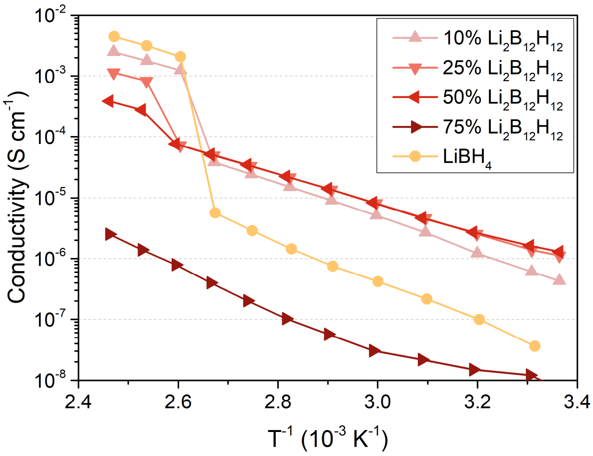

Figure S6. Arrhenius conductivity plots of $LiBH_4$-$Li_2B_{12}H_{12}$ mixtures synthesized at 280 °C. The conductivity of $LiBH_4$ is included for comparison.

S5. Oxidative stability of $LiBH_4$-$LiBF_4$

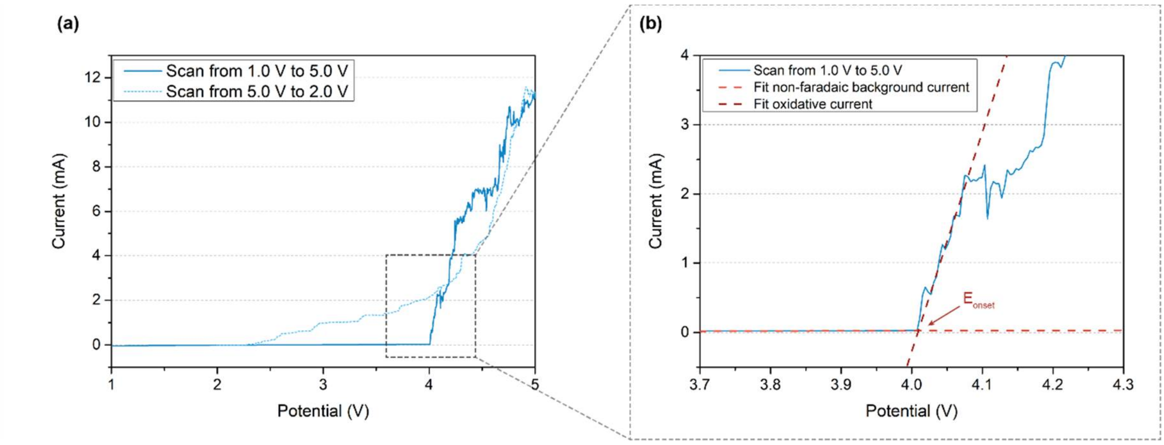

Figure S7. (a) Linear sweep voltammogram of Li|$LiBH_4$-20%$LiBF_4$|$LiBH_4$-20%$LiBF_4$-C|stainless-steel cell at a scan rate of 0.1 mVs-1 from 1.0 V to 5.0 V vs. Li+/Li at 60 C. (b) Determination of the E_{onset} from two linear regression lines of the non-faradaic background current and faradaic anodic current.
